# Systems Biology Approach to the Dissection of the Complexity of Regulatory Networks in the *S. scrofa* Cardiocirculatory System

**DOI:** 10.3390/ijms141123160

**Published:** 2013-11-21

**Authors:** Paolo Martini, Gabriele Sales, Enrica Calura, Mattia Brugiolo, Gerolamo Lanfranchi, Chiara Romualdi, Stefano Cagnin

**Affiliations:** 1 Department of Biology, University of Padova, Via G. Colombo 3, Padova 35121, Italy; E-Mails: paolo.martini@unipd.it (P.M.); gabriele.sales@unipd.it (G.S.); enrica.calura@unipd.it (E.C.); gerolamo.lanfranchi@unipd.it (G.L.); 2 C.R.I.B.I. Biotechnology Centre, University of Padova, Via U. Bassi 58/B, Padova 35121, Italy; E-Mail: brugiolo@mpi-cbg.de

**Keywords:** pathway analysis, miRNA, cardiocirculatory, network reconstruction, integrative analysis, pig, artery, vein, vessel

## Abstract

Genome-wide experiments are routinely used to increase the understanding of the biological processes involved in the development and maintenance of a variety of pathologies. Although the technical feasibility of this type of experiment has improved in recent years, data analysis remains challenging. In this context, gene set analysis has emerged as a fundamental tool for the interpretation of the results. Here, we review strategies used in the gene set approach, and using datasets for the pig cardiocirculatory system as a case study, we demonstrate how the use of a combination of these strategies can enhance the interpretation of results. Gene set analyses are able to distinguish vessels from the heart and arteries from veins in a manner that is consistent with the different cellular composition of smooth muscle cells. By integrating microRNA elements in the regulatory circuits identified, we find that vessel specificity is maintained through specific miRNAs, such as miR-133a and miR-143, which show anti-correlated expression with their mRNA targets.

## 1. Introduction

Genome-wide experiments on RNA expression typically provide lists of differentially expressed genes (DEGs) [1,2] that represent the starting point of a highly challenging process of result interpretation in which the gene-by-gene approach is often used. The lists obtained are highly dependent on the statistical tests adopted and on the threshold used to declare a gene significant. This variability has raised substantial criticism concerning the reproducibility of array experiments. Several studies have demonstrated greater consistency of array results using gene set approaches, rather than single gene approaches [3], indicating that there is greater reproducibility of the main biological themes than of their single elements. A gene set is defined as a set of genes that are functionally related. Gene sets are usually identified based on a priori biological knowledge (see, for example, Gene Ontology “GO” (http://www.geneontology.org/ (accessed on 13 November 2013)) and the Kyoto Encyclopedia of Genes and Genomes “KEGG” (http://www.genome.jp/kegg/ (accessed on 13 November 2013))). In this regard, several new bioinformatics tools have been developed that allow the integration of information such as gene location [4–6], ontological annotations [7–10], or sequence features [11]. These methods can be broadly divided into supervised and unsupervised approaches. Supervised methods use *a priori* information on the functional relationships among genes to identify the processes involved in an experimental condition, while unsupervised approaches attempt to reconstruct functional associations among genes without relying on external information. In the following, we will briefly review these strategies, focusing specifically on their pros and cons; in addition, we will apply these strategies to a case study.

### 1.1. Supervised Approaches: Pathway Analysis

The integration of gene expression profiles with additional information on pathway annotations is called pathway analysis. The pathway analysis approach evaluates gene expression profiles among related genes, looking for coordinated changes in their expression levels. Several implementations of pathway analysis are now available, from the widely used algorithm developed by Subramanian and colleagues (Gene Set Enrichment Analysis; GSEA) [9], with its improvements [10,12], to more sophisticated implementations that exploit the topology of the pathway [13,14] (for a comprehensive review of existing methods, see [15]). Pathway analysis methods can be divided into (i) methods based on enrichment analysis and performed on a list of genes selected through a gene-level test; and (ii) methods based on global and multivariate approaches that define a model based on the whole gene set. With the first class of methods, the primary concerns are the assumption that genes are independent and the use of a threshold value for the selection of differentially expressed genes. Due to the latter, many genes with moderate but meaningful expression changes are discarded based on the strict cut-off value, leading to a reduction in statistical power. On the other hand, global and multivariate approaches relax the assumption of independence among genes belonging to the same gene sets and identify moderate but coordinated expression changes that cannot be detected by the enrichment analysis approach [16].

From this perspective, we recently developed three novel algorithms that can be used to perform gene set and pathway analysis. Graphite, a Bioconductor package [17], is a computational framework that can be used to manage, interpret, and convert pathway annotations to gene-gene networks, while STEPath [18] integrates expression levels and chromosome positioning to identify regional gene activation and CliPPER [14,19] explores the topology of a pathway, highlighting the portions most involved in its deregulation. We have implemented most of these analyses in a new web tool called GraphiteWeb [20].

One of the major drawbacks associated with these approaches is the limitation of pathway annotation. Pathway annotation is a highly challenging procedure that exploits the efforts of many researchers, who manually curate each single pathway based on information available in the literature. Pathways are often thought of as the elementary functional and evolutionary building blocks of the complete metabolic network, with each pathway representing a “self-contained” elementary biochemical process. To partition the reaction network of an organism into a set of (possibly overlapping) metabolic pathways requires arbitrary decisions as to where such partitions should be made and how pathway variants should be described [21]. For these reasons, only a portion (in humans, approximately one-third) of known genes are currently annotated in at least one pathway.

In KEGG [22], the metabolic pathways—called “maps”—are subparts of the overall reaction graph. Reactions within a map are connected by their constituent metabolites, which also provide links to reactions in other maps. KEGG metabolic maps are described without reference to a particular species, and each map includes the reactions belonging to all known variants of a particular pathway. MetaCyc is a database of non-redundant, experimentally elucidated metabolic pathways that are found in many species [23] while, in the smaller Reactome database [24], the human database is used as the reference for predicting reactions and pathways in other organisms.

### 1.2. Unsupervised Approaches: Reverse Engineering Approach

A different approach to dealing with biological networks is the *ab initio* strategy: using genome-wide expression values, these algorithms try to infer the best network of interactions satisfying specific conditions. Unlike the pathway analysis approach, here, all known genes can be taken into consideration. Several methods have been proposed for the reconstruction of gene regulatory networks (GRNs) from experimental data; these include Bayesian Networks (BN) [25], Relevance Networks (RN) [26], and Graphical Gaussian Models (GGM) [27,28]. While BN and GGM distinguish between direct and indirect edges, RN does not. It is worth noting that although BN and GGM are able to infer edge direction this does not necessarily imply an ability to identify biological causality.

BN and GGM function poorly in cases involving thousands of genes and a small number of replicates, while RN has the ability to address such cases. RN uses association measures between two expression profiles, such as correlation and mutual information, to rank gene-gene interactions according to their strengths; the higher the association measure, the greater the probability of a functional interaction between the two genes. All of these approaches produce a large number of false positives (false interactions). The seminal paper of Basso *et al.*, 2005 [29], extends RN, introducing an algorithm based on Data Processing Inequality (DPI) for removing indirect edges. Their approach, called ARACNE (Algorithm for the Reconstruction of Accurate Cellular Networks) [30], has been successfully used to reconstruct the sub-network of the MYC gene in human B cells.

In this context, we developed a new R package, *parmigene*, that performs network inference by implementing an unbiased estimation of the mutual information between expression profiles, thus yielding more precise results than existing software at strikingly less computational cost [31].

Apart from their low specificity, a significant issue raised by the last network inference challenge (DREAM 5) is that no single network inference method performs optimally across all data sets. In contrast, integration of predictions from multiple inference methods through a consensus network shows robust and high performance across diverse data sets [32].

Apart from the algorithm used, once the whole network has been inferred, the classical approach to dealing with large amounts of interactions is identifying small-connected components as a means of testing their enrichment in specific biological processes.

### 1.3. The Missing Element: MicroRNAs (miRNAs)

Although highly innovative, the supervised and unsupervised approaches described so far do not take miRNAs into consideration. Many efforts have been made to predict miRNA/mRNA interactions, first by developing various target prediction algorithms and then by introducing new experimental techniques to isolate miRNA/mRNA complexes [33–36]. Computational target prediction is still widely used, although it is characterized by many false positives. For exhaustive reviews on miRNA discovery algorithms and *in silico* target prediction [37,38].

The integration of target predictions with miRNA and gene expression profiles has recently been proposed as a means of computationally improving and refining miRNA-target predictions. As miRNAs act predominantly through target degradation, the expression profiles of miRNAs and those of their target genes are expected to be inversely correlated [39,40].

Although the key role of miRNA in post-transcriptional regulation is universally recognized, few attempts have been made to use combinations of miRNA elements in developing gene set approaches. The only such attempt was described by Nam and colleagues [41], who performed GSEA on the mRNA targets of de-regulated miRNAs.

### 1.4. Case Study: The Pig as a Model Organism

Considering the advantages and disadvantages of the approaches described above, here we propose a consensus strategy based on the integration of pathway analysis, relevance networks and miRNA expression using as a model organism the pig and its cardiocirculatory system.

The size of organs, as well as various anatomical features, general physiology, and features of organ development, are very similar in pigs and humans. This permits the use of the pig as a model in the study of a number of pathologies, such as those affecting eyes [42], muscle [43], organ transplantation [44,45], and the gastrointestinal [46], nervous [47], and cardiovascular [48] systems. The coronary artery distribution in the pig is more similar to that of humans than is that of other animals. In addition, pigs present very similar cardiac output to humans; they possess a vaso vasorum in the aorta, and the left azygous vein empties into the coronary sinus instead of into the precava. Blood pressure (145–160/105 BP), heart rate (100–150 BPM) and pulmonary pressure are higher in pigs than in humans.

Despite the medical importance of the pig as a species for study, our knowledge of the genome organization, gene expression regulation, and the molecular mechanisms underlying the pathophysiological processes of the pig is far less than the knowledge we have acquired of the mouse and rat. More than 90% of the porcine genome has been sequenced by the Swine Genome Sequencing Consortium [49]. The availability of detailed information on the porcine genome, together with emerging transgenic technologies, will enhance our ability to create specific and useful pig models. Recently, an atlas of DNA methylomes in porcine adipose and muscle tissues was published [50], and a great effort was made to combine genome sequence information with our knowledge of gene expression. Many of these studies focused on the swine immune system [51–54], while a genome-wide expression analysis in different tissues was described in Freeman’s paper [55]. Recently, using sequencing approaches, a compendium of small non-coding RNAs was identified in various pig tissues (e.g., skeletal muscle [56–62], kidney [63], tooth [64], intestinal tract [65], brain [66], testis, ovary, sperm, and embryo [67–71] and pituitary gland [72]). Li and colleagues demonstrated that a complex regulatory network of porcine subcutaneous fat development is reflected in a great diversity of miRNA composition and expression between muscle and adipose tissue [73].

Here, we generate new custom mRNA and miRNA platforms that can be used to dissect the transcriptomic changes and regulatory circuits that are involved in the maintenance of veins and arteries in the pig. An integrative approach, combining pathway analysis and *de novo* network reconstruction, was used to expand our current knowledge of these regulatory circuits and to integrate miRNA activity into these circuits demonstrating their role in vessel specification. We show that vessel specificity can be maintained through different miRNAs (e.g., miR-133a and miR-143), the expression of which is inversely correlated with that of their mRNA targets.

## 2. Results and Discussion

The integration and analysis of gene and miRNA expression profiles across different tissues is fundamental to our understanding of tissue-specific processes. Here, we focus our analysis on differences in gene and miRNA expression among different tracts of the circulatory system: the two largest veins of the body (superior and inferior vena cava), the aorta (ascending and descending), the pulmonary artery, and the coronary artery. To achieve this goal, we created mRNA and miRNA [74] platforms, the latter based on the RAKE (RNA primed–array-based Klenow enzyme assay) method [75,76], to quantify coding and non-coding gene expression in pig tissues. After quantifying miRNA and mRNA expression, we used a combination of supervised and unsupervised approaches to detect transcriptional and post-transcriptional differences among different tracts of the circulatory system.

Ensembl transcripts (Ver. 56; EMBL-EBI, Wellcome Trust Genome Campus, Hinxton, Cambridgeshire, UK) and UniGene (Ver. 38; National Center for Biotechnology Information, U.S. National Library of Medicine, Bethesda, MD, USA) pig sequences were used to produce a dedicated microarray platform for monitoring mRNA expression. On the basis of sequence similarity, UniGene features that overlapped more than 40% with an Ensembl transcript were discarded. After this filter, we obtained 40,267 UniGene clusters and 19,603 Ensembl transcripts (protein coding + pseudogenes + retrotransposed elements). For this selected collection of sequences, we designed microarray probes with different specificities and located at different distances from the 3′ ends of specific transcripts using six different algorithms. The two best probes for each sequence, as determined by the reliability of the prediction algorithm and by the probe’s vicinity to the 3′-end, were experimentally tested in a hybridization trial performed with a pool of mRNA populations independently prepared from 20 pig tissues (GEO: GSE28636). For each transcript with a replicated probe, we selected the probe that was the most responsive and specific on the basis of the intensity of fluorescence in the hybridization test, as suggested by Kronick [77]. The resulting pig whole-genome microarray, which was used in the gene expression analysis, is composed of: (i) 17,048 replicated probes and 963 single probes specific for the Ensembl transcripts; (ii) 11,363 replicated probes specific for the UniGene clusters of lengths between 778 nt and 1348 nt; and (iii) 28,790 single probes specific for the remaining UniGene clusters. Our analysis was not able to identify specific probes for 114 UniGene clusters and 1592 Ensembl transcripts. A limitation we faced in working with gene expression in pig was the poor gene annotation available. The number of annotated features on the array was increased by mining description and protein annotations to associate gene names with our probe symbols. Basically, for genes for which the HUGO (Human Genome Organisation) symbol was not present, we mined the description available from the Unigene database and retrieved additional gene or protein IDs, if present. All IDs were manually curated (ArrayExpress ID: A-MEXP-2351).

Recently, a new microarray platform based on 52,355 expressed sequences comprising miRNAs in miRBase Ver. 15 (Wellcome Trust Sanger Institute, Cambridge, UK) for pigs, cows, humans, and mice was described [55]. Unlike this new platform, which was constructed by spanning 22 probes along the transcripts, the platform we developed detects the 3′-UTR of each transcript; therefore, we are able to distinguish mRNA isoforms. This feature is fundamental because the activity of miRNAs is predominantly based on their interactions with the 3′-UTR region of mRNAs.

**Figure 1:**
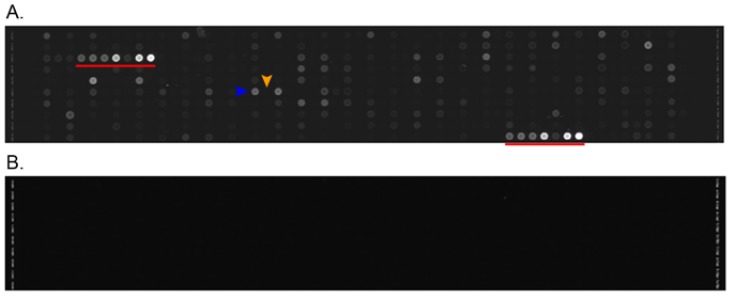
Explicative scan portion of miRNA microarray after the RAKE and labeling reactions (**A**) and before hybridization (**B**). Spike-in spots are indicated by red lines; the blue arrow indicates a specific probe, and the orange arrow indicates its background probe. Each background probe was positioned to the right of its probe.

The identification of miRNAs was described in [74]. Briefly, bioinformatic analyses were performed on the pig genome for the identification of putative pre-miRNAs. These were experimentally tested using six independent RAKE experiments to identify 5′ and 3′ miRNA boundaries. After this experimental confirmation, all the pre-miRNAs identified as responsive (1235 hairpins) were tested for the presence of mature miRNA through RNA sequencing experiments. RNA sequencing experiments identified 343 hairpins coding for miRNAs. However, using PCR we were able to validate several miRNAs that were not confirmed by RNA sequencing. Therefore, we decided to produce an miRNA microarray platform (Array Express ID: A-MEXP-2348) containing all miRNAs detected by RAKE experiments. In the following analysis, we will discuss only miRNAs that were confirmed in sequencing experiments. Each specific probe is flanked by a background probe that was used to subtract the corresponding background fluorescence signal in the analysis (Figure 1).

The short length of miRNAs makes complementary probe selection and the identification of optimized PCR primers a challenging task. While miRNA microarrays permit massive parallel and accurate relative measurement of all known miRNAs, they have been less useful for absolute quantification. We developed a new method that integrates the hybridization of miRNAs with an enzymatic elongation reaction that can take place only following a perfect match between the miRNA and the probe. Moreover, we introduced oligonucleotide spikes into the hybridization-enzymatic reaction, permitting the quantification of miRNAs over the linear dynamic range of 10^−18^ moles to 10^−14^ moles and avoiding biases related to sequence, labeling, or hybridization [74].

### 2.1. Differences between Arteries and Veins

**Figure 2:**
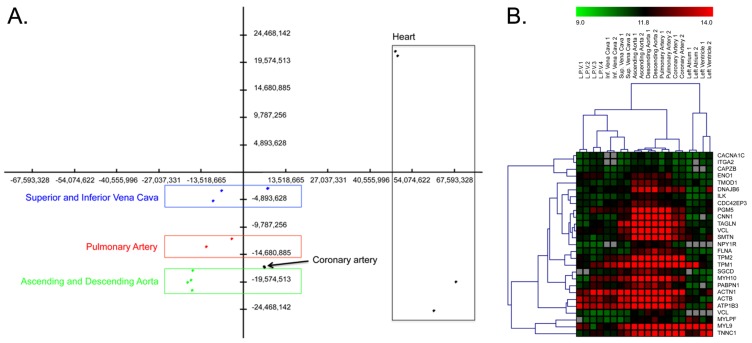
(**A**) Principal component analysis (PCA). The first three components account for 62.8% of the observed variance. The green rectangle identifies the group of ascending and descending aorta samples (green dots); the coronary artery is indicated by a black dot, the red rectangle highlights pulmonary artery samples (red dots), and the blue rectangle surrounds superior and inferior vena cava samples (blue dots). On the right, separated from other samples, are heart samples; (**B**) Heat map of muscle transcripts. Transcripts coding for muscle proteins are up-regulated in arteries with respect to veins. The red squares indicate up-regulated genes, and the green squares indicate down-regulated genes. The grey squares indicate genes for which no expression was detected. L.P.V. = leaflet of pulmonary valve; Inf. Vena Cava = inferior vena cava; Sup. Vena Cava = superior vena cava. The numbers following the sample names indicate the number of experimental replicates.

We compared different tracts of the circulatory system: the two largest veins (the superior and inferior vena cava), the aorta (ascending and descending tracts), the pulmonary artery, and the coronary artery. As expected, the ascending and descending aorta and the coronary artery display similar gene expression profiles that are distinct from those of the superior and inferior vena cava (Figure 2A), while the pulmonary artery has an intermediate expression profile (Figure 2A). Arteries and veins are structurally different in terms of their relationship to the heart. Arteries receive blood directly from the heart and are therefore characterized by high pressure; in contrast, veins receive blood from peripheral body regions, and low pressure characterizes them. For this reason, some of the blood in the veins may not return to the heart but instead may back up or collect in these vessels. Veins transport de-oxygenated blood, while arteries transport oxygenated blood (with the exception of the pulmonary artery, which transports de-oxygenated blood to the lungs for oxygenation). The difference in blood pressure in arteries and veins is reflected in the different structures of these vessels. Arteries and arterioles have thicker walls than veins and venules; specifically, they possess an increased amount of smooth muscle that provides extra strength and elasticity to withstand surges of blood from the heart. Moreover, the thinner the vessel, the lower its innervation.

In accordance with the increased number of smooth muscle cells in arteries, the aorta expresses more smooth muscle-specific transcripts than the vena cava (Figure 2B). Genes that are up-regulated in the aorta include genes related to biological structures such as adherence junctions and processes such as nerve function and blood circulation (Table S1). This is consistent with the significantly higher level of innervation of arteries than of veins. Up-regulated genes in the vena cava are enriched in genes coding for proteins involved in the formation of the extracellular matrix (Table S1). These findings may be associated with the differences in elasticity between veins and arteries (veins have less elastic tissue than arteries).

A major component of the vessel walls of large arteries and veins is the extracellular matrix (ECM), which consists of collagens, elastin, and proteoglycans. The smooth muscle cells of the aorta and vena cava synthezise different amounts of collagen. As expected, our data show that collagen synthesis is four-fold higher in venous than in arterial [78]; collagen type I (COL1A2) is the most highly expressed extracellular matrix component.

*Procollagen C-endopeptidase enhancer 2* (*PCOLCE2*) and *P4HA1 prolyl 4-hydroxylase*, *α polypeptide I* (*P4H4*) genes were found to be up-regulated in the vena cava. PCOLCE2 binds to the *C*-terminal propeptide of type I and II procollagens and may enhance the cleavage of their propeptides, while P4H4 is a key enzyme in collagen synthesis. Moreover, we found *type VIII collagen* (*COL8A1*), which is typical of the endothelium lining vessels, and *type VI collagen* (*COL6A3*), a subendothelial constituent [79], to be highly expressed in the vena cava.

### 2.2. Pathway Analysis

Using multivariate pathway analysis methods such as GSEA, we overcame the major limitation of the classical enrichment approach, cut-off-based gene selection, focusing instead on coordinated changes in gene expression. Using this method, we were able to identify gene pathways that are specifically expressed in arteries and veins (Table 1). Among the activated pathways in arteries are those associated with smooth muscle contraction, calcium-calmodulin-dependent events, genome stability and regulation of intracellular signaling cascades. This finding is consistent with the presence of a thicker smooth muscle ring in arteries than in veins. Among the activated pathways in veins, we find the complement cascade, arachidonic acid metabolism, cell surface interactions at the vascular wall, and extracellular matrix metabolism (glycosaminoglycan metabolism and keratin/keratan sulphate metabolism). Arachidonic acid metabolism is involved in the control of various processes within the cardiocirculatory system, including vasoconstriction [80] and vasodilation [81,82]. The two most highly expressed genes related to arachidonic acid metabolism were *prostaglandin-endoperoxide synthase 2* (*PTGS2 or COX-2*) and *γ-glutamyltransferase 5* (*GGT5*). *COX-2* and *endothelial nitric oxide synthase* (*eNOS*) are primarily expressed in endothelial cells and are considered important regulators of vascular function. Under normal conditions, laminar flow induces COX-2 expression and synthesis of PGI_2_, which in turn stimulates eNOS activity [83]. GGT expression was also localized in the endothelium [84]. As blood normally flows more slowly through veins than through arteries, thromboses are more common in veins than in arteries. This could be the reason for the control of vasodilation and vasoconstriction through metabolites of arachidonic acid.

**Figure 3:**
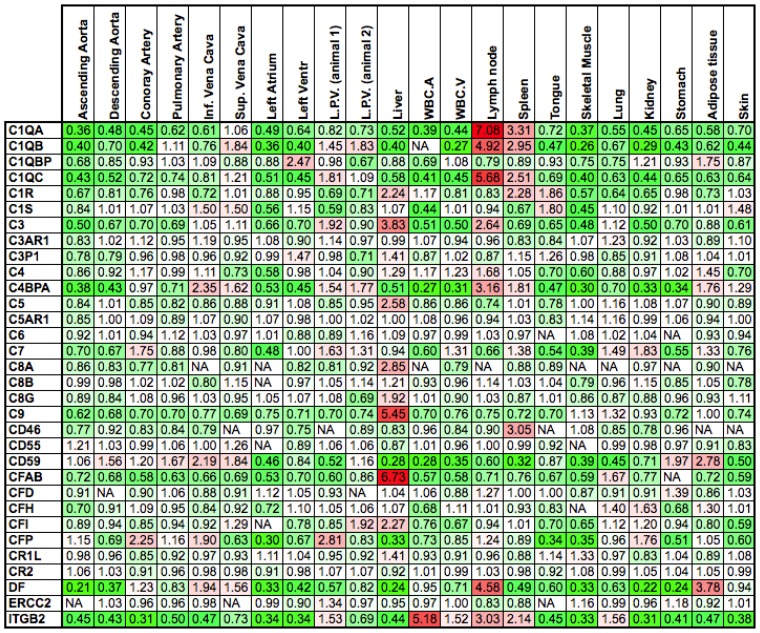
Expression of genes involved in the complement response. The numbers represent gene expression levels normalized to the average expression of the same gene across all tissues. Down-regulated genes are shown in green, and up-regulated genes are shown in red. Most of the up-regulated genes are expressed in the liver, which is responsible for the synthesis of most of the proteins of the complement system, in the spleen and in lymph nodes (lymphoid organs). NA = Expression not detected; L.P.V. = leaflet pulmonary valve; WBC.A = white blood cells from arterial blood; WBC.V = white blood cells from venous blood.

In support of the up-regulation of elements of the complement cascade in veins, it is known that inflammation is more readily induced in venous than in arterial epithelium due to the conditions of the venous circulation. We checked for the presence of an inflammatory process by analyzing the expression of complement components in 19 tissues (Figure 3). We find that not all complement components are up-regulated in veins, while most are highly expressed in lymph nodes, spleen, and liver. This is in accordance with complement system synthesis and laundering. The complement system consists of a dozen circulating proteins, most of which are synthesized by the liver, that have the ability to bind to cellular membranes. The spleen and the liver are able to remove immune complexes composed of complement elements linked to erythrocyte membranes [85].

Finally, it is worth noting that pathways describing mucopolysaccharidosis syndromes such as Hurler, Sanfilippo, and Morquio syndromes were found to be significantly expressed in veins. Altered glycosaminoglycan metabolism is a key feature of these pathologies. Glycosaminoglycans are proteoglycans that bind to a varying degree water, electrolytes and macromolecules, such as collagen, within the connective tissue. The lining of veins and arteries comprises a substantial amount of the body’s connective tissue. The outer layer of vessels (tunica adventitia) consists chiefly of connective tissue and is the thickest layer of the vein.

### 2.3. *De Novo* Pathway Reconstruction: Topological Parameters

**Figure 4:**
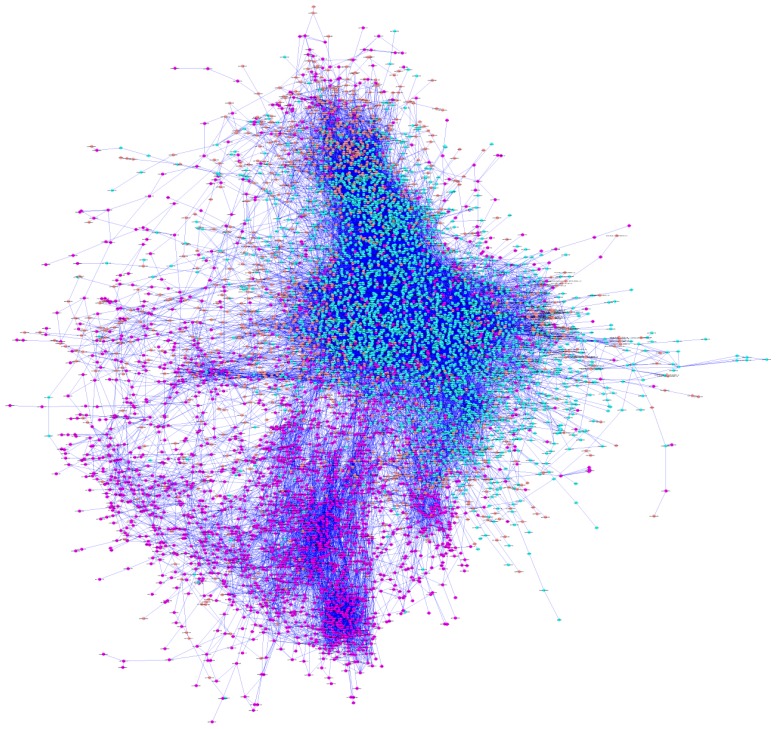
Regulatory network reconstructed using mutual information. The edges of the network are colored according to their prevalent expression. Heart-specific genes are shown in violet, vessel-specific genes are shown in blue, and genes without tissue-specific expression are shown in pink.

Pathway analysis fails to consider many known genes and miRNAs that are not annotated in any pathway. To fill these gaps, we used *de novo* network reconstruction using both mRNA and miRNA profiles. Using a correlation measure with a permutation-based threshold of 0.9 of mutual information (0.9 was the maximum value of mutual information of the network generated by the permuted expression matrix), we generated a network with 7762 nodes (7647 genes and 115 miRNAs) and 44,092 edges (Figure 4). The global architecture of the network is characterized by two large clusters, which are shown as the blue and violet nodes in Figure 4. As expected (Figure 2A), these two clusters are composed of genes prevalently expressed in heart (the most different tissue) and in blood vessels (Figure S1). Thus, we separated these two clusters to create a vessel-specific and a heart-specific network.

To gain insight into the structure of complex networks of this type, various topological parameters were calculated (Table 2). The heart network is sparser and less connected than the vessel network. This is reflected by a larger number of connected components, a higher diameter and a smaller number of neighborhood genes of the heart network.

The degree of a node, also referred to as its connectivity, is the number of edges connected to the node. Based on this definition, the nodes with the highest connectivities are called hubs. In general, hub genes are master regulators and play important roles in the biology of the cell. In our networks, we define as hubs the top 5% of genes in the connectivity distribution. We found 162 and 128 hubs in the vessel and heart networks, respectively. The hub genes of the vessel network encode proteins that participate in two main processes: RNA processing and the regulation of apoptotic events (Table S2). During normal development as well as in pathology, the formation of new vessels and the regression of pre-existing ones depend on the balance between endothelial cell proliferation and endothelial cell apoptosis. In mature vessels, endothelial cell turnover is also under the control of these tightly regulated phenomena. Among the hubs of the heart network, we identified genes involved in cell membrane structure and signal transduction through MAPK activity as well as genes encoding various ion transporters (e.g., Na^2+^, K^+^) (Table S2). The members of the MAPK family are involved in the regulation of many cellular processes, including cell growth, differentiation, development, the cell cycle, death, and survival. Activation of genes in the MAPK family plays a key role in the pathogenesis of various processes in the heart, including myocardial hypertrophy and its transition to heart failure, ischemic and reperfusion injury, and cardioprotection conferred by ischemia- or drug-induced preconditioning [86].

**Figure 5:**
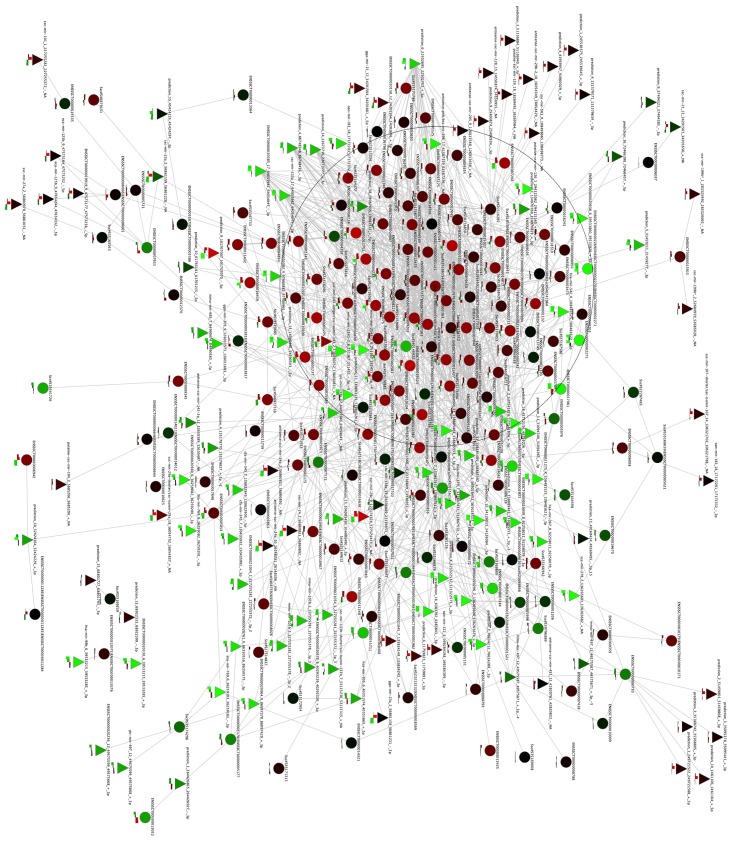
Gene and miRNA interaction sub-network describing vessel specificity. Triangles represent miRNAs; circles represent mRNAs. Gene expression in the ascending aorta according to log_2_ (gene expression/average gene expression) is represented by color; green indicates down-regulation, red indicates up-regulation. Under each node, histograms representing log_2_ (gene expression/average gene expression) in the ascending aorta, descending aorta, inferior vena cava, and superior vena cava (reading from left to right) are shown. The area highlighted by the circle indicates the densely connected portion of the sub-network (an enlarged view of this area is available in Figure 6).

**Figure 6:**
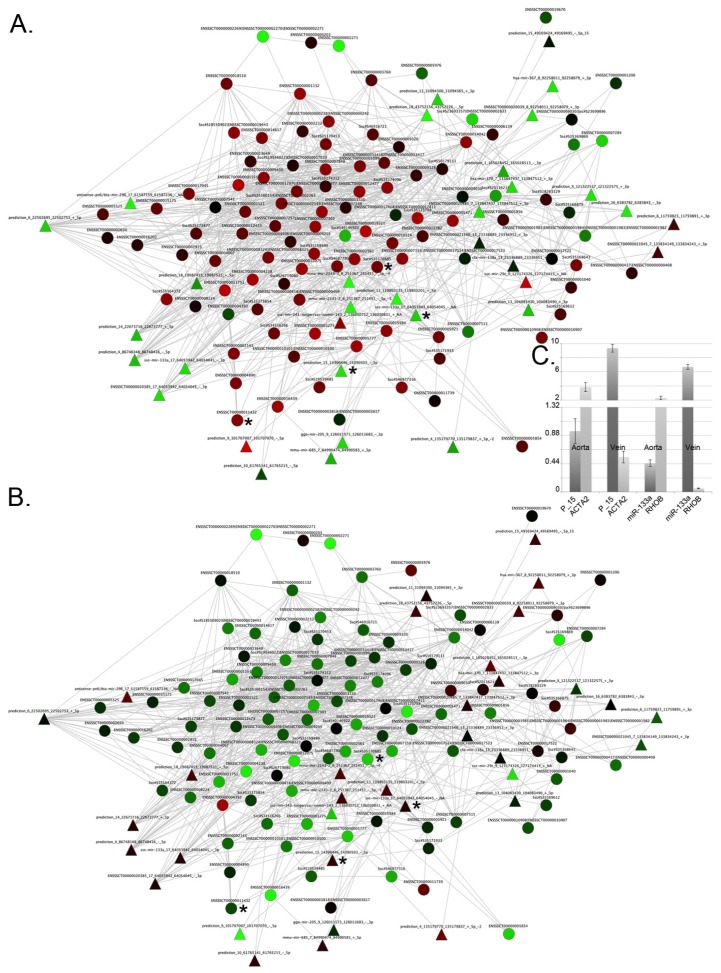
Enlarged view of the densely connected area of Figure 5. (**A**) The colors indicate expression in the aorta; (**B**) The colors indicate expression in veins. The triangles represent miRNAs; circles represent mRNAs. Up-regulated = red; down-regulated = green; * = nodes discussed in the text; (**C**) qRT-PCR results confirm that there is an inverse relationship between miRNAs and their targets. P_15 is for prediction_15_14390446_14390503_-_3p. In *Y* axis the original expression level related to H3. Bars are for standard deviation between three replicates.

The *de novo* reconstructed network (Figure 4) is characterized by the presence of different miRNAs (Table S3) that are responsible for the regulation of vessel specificity. Figure 5 represents the sub-network of the neighboring genes of miRNAs. Interestingly, the central part of the network (the densely connected portion of the sub-network) is characterized by genes involved in smooth muscle contraction (Table S4) that show differential expression in arteries and veins (Figure 6). As discussed previously, a thicker ring of smooth muscle is present in arteries than in veins (see Section 2.2). Our results suggest that this difference may be regulated by specific miRNAs that display anti-correlated expression with their putative targets (Figure 6).

Specifically, the *α 2-actin* (ACTA2) smooth muscle gene in aorta (ENSSSCG00000010447) is regulated by a specific miRNA (prediction_15_14390446_14390503_-_3p) that is down-regulated in the aorta and up-regulated in venous tissue (Figure 6). Defects in ACTA2 are the cause of aortic aneurysm familial thoracic type 6 (AAT6) [MIM:611788]. AATs are characterized by permanent dilation of the thoracic aorta, usually due to degenerative changes in the aortic wall. RHOB (Ssc#S35170885), an important gene involved in vasoconstriction, is also regulated by miR-133a (Figure 6). RHO gene family is involved in vascular morphogenesis [87], and miR-133a contributes to the phenotypic state of smooth muscle cells both *in vitro* and *in vivo*, suggesting a potential for therapeutic application of this miRNA in vascular disease [88]. In fact, miR-133a, in association with miR143/145, is fundamental for the maintenance of the contractile smooth muscle cell phenotype [88]. The expression of miRNAs prediction_15_14390446_14390503_-_3p and miR-133 and their targets ACAT2 and RHOB was confirmed by qRT-PCR (Figure 6C).

### 2.4. Integration of Supervised and Unsupervised Approaches

**Figure 7:**
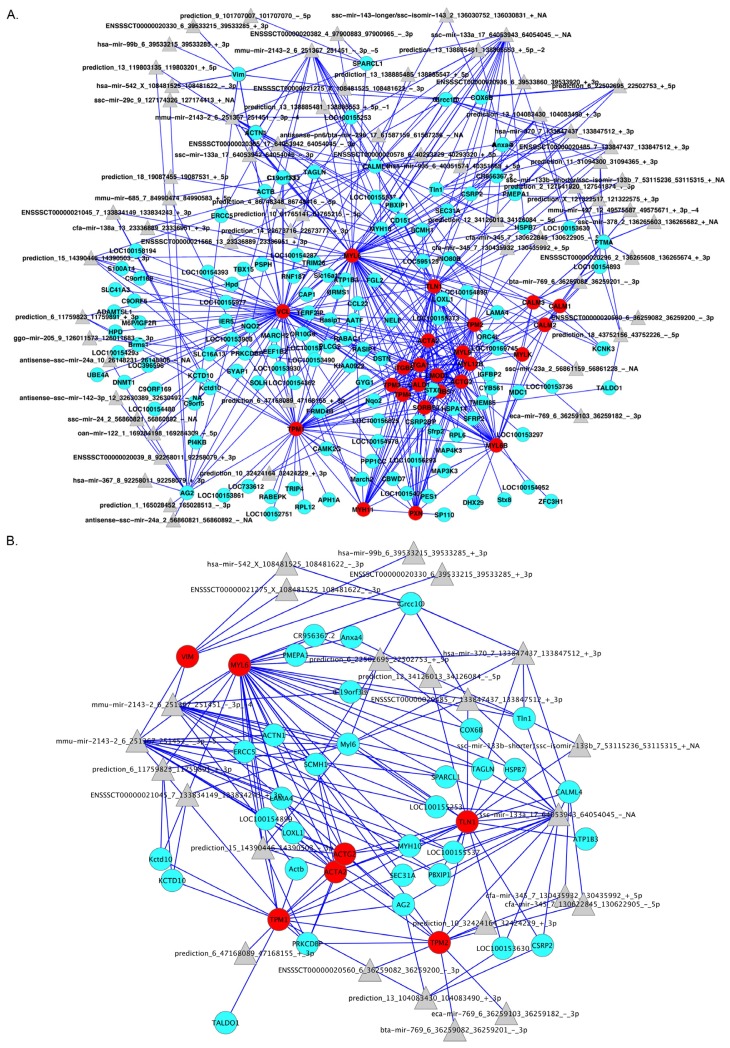
(**A**) Combination of pathway topology and *ab initio* reconstructed network. Nodes corresponding to the Reactome pathway (core nodes) are shown in red; additional genes in the first neighborhood of the core nodes obtained from the *ab initio* network are shown in light blue, and miRNAs are shown in grey; (**B**) Portion of (**A**) representing the miRNAs regulating the core nodes.

Supervised and unsupervised approaches gave similar results in terms of biological processes involved in tissue specificity. However, their complementary behavior might be better exploited through the use of an integrative approach. Specifically, our aim is to combine the topology of the discovered pathways with that of the *de novo* reconstructed network. The advantage of combining the topologies obtained in sections 2.2 and 2.3 is two-fold: (i) it allows the expansion of pathway definitions to include genes currently without pathway annotation; and (ii) it permits the inclusion of miRNAs. Using the topological structure of the pathway as a backbone, we include new genes in the pathway, following two rules: (i) a gene/miRNA is added only if it presents an edge in the *de novo* network with at least one gene in the pathway; and (ii) additional miRNAs are included if they share an edge with previously added non-annotated genes. Here, we will use this strategy to discuss one of the most interesting pathways significantly activated in arteries: the smooth muscle contraction pathway (Figure 7A). The genes used to expand this pathway (the γ isoform of the catalytic subunit of *protein phosphatase 1* (*PPP1CC*), *transgelin* (*TAGLN*), and smooth muscle and non-muscle *myosin light chain 6* (*Myl6*), among others) are primarily involved in membrane and actin filament organization, actomyosin function and responses to specific stimuli (NF-κB binding and response to unfolded protein) (Table S5), reflecting their functional congruence with the smooth muscle contraction pathway. Indeed, the membrane organization category includes the organismation of the sarcoplasmic reticulum, which is involved in the regulation of intracellular Ca^2+^ concentration (Figure 7). All of these genes are prevalently expressed in smooth muscle; in particular, TAGLN was purified from bovine aorta [89]. Moreover, we added 61 miRNAs that putatively regulate genes involved, directly or indirectly in the smooth muscle contraction pathway (Figure 7A). Interestingly, 23 miRNAs are involved in the regulation of the original genes of the pathway (core genes). Among these miRNAs, miR-542 (ENSSSCT00000021275), which was shown in a previous work to be involved in the epithelial-mesenchymal transition [90], was found to be associated with *vimentin* (*VIM*) regulation (Figure 7B). Finally, it is worth noting that many other miRNAs important for vascular remodeling and smooth muscle phenotypic control, such as miR-133 [88], miR-143 [91], miR-99b [92], miR-23a [93], miR-138 (ENSSSCT00000021566) [94], miR-29c [95], miR-125a (ENSSSCT00000020936) [95], and miR-24 [96]), are included in this network.

## 3. Experimental Section

### 3.1. Sample Preparation

RNA samples (total RNA and small RNAs) were extracted from the analyzed tissues of three non-inbred pigs and kept at −80 °C until use. Before the experiments were performed, the three samples from the same tissues were pooled, and miRNA was selected using a flashPAGE instrument (Ambion, Carlsbad, CA, USA). RNA extraction was performed using TRIzol (Invitrogen, Carlsbad, CA, USA) according to the manufacturer’s protocol. The PureLink Isolation Kit (Invitrogen, Carlsbad, CA, USA) was used to separate long RNA from short (<200 nt, after use in the flashPAGE instrument). All samples were quantitated using a NanoDrop ND-1000 spectrophotometer; RNA quality was then analyzed using the Agilent Bioanalyser 2100 (Agilent, Santa Clara, CA, USA) (Agilent RNA 6000 nano kit; RIN at least 7 accepted) and for the presence of miRNA using the Agilent small RNA kit.

### 3.2. Microarray Platforms

For this study, we synthesized two different types of microarray platforms: (a) 4 × 2 K Combimatrix microarrays for miRNA expression profiling (ArrayExpress ID: A-MEXP-2348); (b) 90 K Combimatrix microarrays (ArrayExpress ID: A-MEXP-2351) for mRNA expression profiling. All microarrays were synthesized using the Combimatrix oligonucleotide synthesizer station (Combimatrix, Mukilteo, WA, USA), which allows *in situ* synthesis of oligonucleotide probes through phosphoramidite chemistry. All synthesized microarray platforms were tested for uniformity of the probes as suggested by the manufacturer.

The 4 × 2 K microarrays contain specific probes for miRNAs. Each specific probe is flanked by a background probe that is used in the analysis to subtract the corresponding background fluorescence signal (Figure 1). The background probes were derived from a previous RAKE experiment aimed at the identification of specific ends of miRNAs in which a tiling microarray was used for the scope (Figure S2) [74].

### 3.3. Microarray mRNA and miRNA Gene Expression and qRT-PCR

#### 3.3.1. mRNA

Pooled RNA (1 μg; three samples from the same tissue) was linearly amplified and labeled by the addition of biotinylated nucleotides according to the procedure described in the Ambion MessageAmp™ II aRNA Amplification kit (Ambion, Carlsbad, CA, USA). The procedure includes reverse transcription with an oligo-dT primer carrying a T7 promoter to produce the first-strand cDNA. After second-strand synthesis and clean-up, the cDNA is used as template in an *in vitro* transcription reaction to generate a large quantity of antisense RNA (aRNA). Biotinylated UTPs were incorporated into the aRNA during the *in vitro* transcription reaction. Following purification, 18 μg of aRNA was fragmented using the Ambion Fragmentation Kit (Ambion, Carlsbad, CA, USA). Intact and fragmented aRNAs were tested on an Agilent Bioanalyzer 2100 (Agilent, Santa Clara, CA, USA) using the RNA 6000 Nano LabChip (Agilent, Santa Clara, CA, USA). The size of intact aRNAs ranged from 300 to 4000 nucleotides, while that of fragmented aRNAs ranged from 50 to 250 nucleotides. Fragmented aRNA was hybridized to pre-hybridized 90 K Combimatrix microarrays. The pre-hybridization step was performed for 2 h at 42 °C in a solution containing 5× Denhardt’s solution, 100 ng/μL salmon sperm DNA and 0.05% SDS in 1× Hyb solution prepared as suggested by Combimatrix. Hybridizations were carried out with 4.8 μg of fragmented aRNA in 25% DI formamide, 100 ng/μL salmon sperm DNA and 0.04% SDS in 1× Hybridization solution at 42 °C for 18 h with constant mixing. After hybridization, the microarray platforms were washed with the following:

- 6× SSPET (SSPE added with 0.05% of Tween-20) preheated at 42 °C for 5 min;
- 3× SSPET for 1 min at room temperature;
- 0.5× SSPET for 1 min at room temperature; and
- PBST for 1 min at room temperature.

The microarray chamber was then filled with biotin blocking solution (0.1% Tween-20 and 10 mg/mL BSA in 2× PBS) and incubated at room temperature for 1 h. Labeling was performed by incubating the microarray with dye labeling solution (0.1% Tween-20, 10 mg/mL BSA and 1.6 ng of Cy3-streptavidin (Amersham, Little Chalfont, UK) in 2× PBS) for 1 h at room temperature. After the washing steps (PBST for 1 min at room temperature two times; PBS for 1 min at room temperature), microarrays were scanned at 3 μm resolution with the VersArray ChiprRaderTM (BioRad, Hercules, CA, USA) (ArrayExpress ID: E-MTAB-1941).

#### 3.3.2. miRNA

A sample of the miRNA pool (350 ng) was hybridized for 20 h at 37 °C in a static hybridization oven in hybridization buffer consisting of 6× SSPE, 8 mg/mL BSA, 700 ng of small RNAs and spike-in. After hybridization, the microarrays were washed with the following stringent procedure:

- 1 min at room temperature with 6× SSPET (SSPE containing 0.05% Tween-20);
- 1 min at room temperature with 3× SSPET;
- 1 min at room temperature with 2× PBS;
- 1 min at room temperature with 1× Buffer 2 (the buffer for the Klenow enzyme).

The RAKE reaction was performed at 36.5 °C by incubating the microarray for 1.5 h in 1× Buffer 2 containing 16 μM biotin-14-dATP (Invitrogen, Carlsbad, CA, USA) and 0.25 U/μL Klenow fragment (3′→5′ exo−) (NEB, Ipswich, MA, USA). The microarrays were washed two times in 1× Buffer 2 and incubated in biotin blocking solution for 1 h at room temperature. Extended miRNAs (primers) were labeled by incubating the microarray in the dye labeling solution for 1 h at room temperature. The microarrays were rinsed in PBST (0.1% Tween-20 in 2× PBS) for 1 min at room temperature and in 2× PBS for 1 min at room temperature and scanned (ArrayExpress ID: E-MTAB-1938).

qRT-PCR was used to validate the expression of miRNAs and mRNAs. For mRNA, the SYBR green approach was used in association with the *Power* SYBR^®^ Green PCR Master Mix (Applied Biosystems, Carlsbad, CA, USA); for miRNA, the NCode™ SYBR^®^ Green miRNA qRT-PCR Kit (Life Technologies, Carlsbad, CA, USA) was used according to the manufacturer’s specifications. The primers used were GCATGCAGAAGGAGATCACA (left) and GCTGGAAGGTGGACAGA GAG (right) for ACTA2, TATGTGCTTCTCGGTGGACA (left) and CGAGGTAGTCGTA GGCTTGG (right) for RHOB, and GGTTCCCAGGCTAGGGGTCG (specific) for prediction_15_14390446_14390503_-_3p and CAGCTGGTTGAAGGGGACCA for miR-133a. The reference genes used were GAPDH for mRNA and snU6 for miRNA. The results shown are normalized to the expression of histone H3.

### 3.4. Data Analysis

Images of hybridized mRNA microarrays were quantitated using the Combimatrix imaging software. The raw data were normalized using the quantile method. The goal of the quantile method is to normalize the distribution of probe intensities across a set of microarrays. After normalization, the fluorescence intensities of probe spots presenting values lower than the average of the medians of all negative control probes were set as missing values (NA). The negative control probes were used to calculate the background value (filter). Probe spots presenting NA in more than six experiments were excluded from data analysis. Before performing the analysis, the intensity values of the replicated probes were averaged. Differentially expressed genes were identified using the MeV suite [97] and applying PCA (Principal Components Analysis) [98] and SAM (Significance Analysis of Microarrays) [2] analysis. COA (Correspondence Analysis) analysis [99] was used to determine the specificity of the *de novo* reconstructed network. Gene enrichment was performed using the DAVID web application [100]; pathway analysis was performed using GraphiteWeb [20].

miRNA data were pre-processed as previously described except that cyclic lowess normalization was applied [101]. After inter-array normalization, the fluorescence intensity of the specific miRNA probe was subtracted from the corresponding background fluorescence and used to extrapolate the miRNA concentration from the spike-in-derived curve. The spike-in curve was extrapolated using spline interpolation [102].

Pig gene symbols from Ensembl were converted to human gene symbols using the Ensembl orthologous database through the BioMart service. For UniGene clusters, we extracted the most similar protein or gene curated by NCBI (http://www.ncbi.nlm.nih.gov/ (accessed on 13 November 2013)) based on sequence similarity and then used the NCBI HomoloGene database to translate the protein or gene to its human homolog. This method is commonly used to map genes to pathways in non-model organisms or to map genes that are poorly annotated in model organisms [103]; it is also common to use the well-curated human pathways to extrapolate pathways for non-model organisms. GSEA [10] was then performed using the GraphiteWeb web tool [20].

Mutual information (MI) between all pairs of genes and miRNAs was estimated using the parmigene Bioconductor package [31]; miRNA-miRNA interactions have been removed. To assess MI significance, we estimated the null distribution using a permutational approach. The expression profiles of miRNAs and mRNAs were randomly shuffled, and MI was then estimated on the shuffled matrices. To generate the global network, we included only interactions with MI that were greater than the maximum MI value obtained from the null distribution, which was 0.9 (corresponding to quantile 0.999 in the empirical distribution).

The Cytoscape tool [104] with the Networkanalyser [105] plugin was used to estimate the topological properties of heart and vessel networks.

The topologies of the most interesting pathways derived from pathway annotation (graphite Bioconductor package) were integrated with the topology of the *de novo* reconstructed network. The combination was performed using the pathway topology as backbone; new genes/miRNAs were then added based on fulfilment of one of the following criteria: (i) if the new gene/miRNA shares an edge in the *de novo* network with at least one gene in the pathway; and (ii) if an miRNA shares an edge in the *de novo* network with at least one previously added gene.

## 4. Conclusions

Gene set analyses have been shown to provide better insights and more robust results in array experiments than classical gene-by-gene approaches. Here, we reviewed various strategies used in gene set analysis and showed how to address their integration. We combined genome and pathway information with expression data and applied this approach to a case study, the analysis of the pig cardiocirculatory system. Two new platforms for pig transcriptome analysis (mRNA and miRNA) were presented and applied to the study of tissue specificity. Different expression patterns were identified in heart and vessels; within these, arteries show distinct profiles from those of veins. These findings seem to be associated with the functional and structural composition of the vessels. In agreement with histochemical evidence, pathway analysis revealed the greater importance of smooth muscle in arteries than in veins. We showed that miRNAs participate in the definition of arterial and venous pathways; specifically, for smooth muscle, our data indicate the importance of miR-133a in regulating the *RHOB* gene. The use of a combination of supervised and unsupervised approaches allowed us to expand the compositions of known pathways to include new genes involved in membrane and actin filament organization, actomyosin function and response to stimuli and new miRNAs, most of which are known to be associated with vascular remodeling and control of the smooth muscle phenotype. These results demonstrate the feasibility and usefulness of combining these two approaches in identifying new candidate genes whose expression is associated with specific experimental conditions.

## Supplementary Information



## Figures and Tables

**Table 1:** Summary of Gene Set Enrichment Analysis (GSEA) analysis based on the Reactome database (http://www.reactome.org/ (accessed on 13 Novembre 2013)). Set size refers to the dimension of the pathway, and NTK (Normalized *T*-test of the kth gene set) is the observed value of the statistic as defined in the Graphite web tool [20]. Negative NTK values indicate pathways activated in veins, while positive values indicate pathways activated in arteries. It is worth noting that GSEA is known to have low statistical power; the suggested *Q*-value cut-off for identification of significant pathways is 0.25.

Pathway	Set size	NTk	*Q*-Value
Complement cascade	18	−5.29	0
Arachidonic acid metabolism	11	−3.09	0.044912281
Glycosaminoglycan metabolism	54	−3.09	0.044912281
MPS I—Hurler syndrome	54	−3.09	0.044912281
MPS II—Hunter syndrome	54	−3.09	0.044912281
MPS IIIA—Sanfilippo syndrome A	54	−3.09	0.044912281
MPS IIIB—Sanfilippo syndrome B	54	−3.09	0.044912281
MPS IIIC—Sanfilippo syndrome C	54	−3.09	0.044912281
MPS IIID—Sanfilippo syndrome D	54	−3.09	0.044912281
MPS IV—Morquio syndrome A	54	−3.09	0.044912281
MPS IV—Morquio syndrome B	54	−3.09	0.044912281
Biological oxidations	56	−2.75	0.106666667
Cell surface interactions at the vascular wall	54	−2.75	0.106666667
Keratan sulfate/keratin metabolism	20	−2.46	0.205977011
G α (12/13) signaling events	35	−2.37	0.24
Antigen presentation: Folding, assembly and peptide loading of class I MHC	11	−2.33	0.250980392
Golgi associated vesicle biogenesis	29	−2.29	0.247017544
Glutathione conjugation	10	−2.26	0.249756098
Phase II conjugation	23	−2.26	0.249756098
EGFR interacts with phospholipase C-γ	17	2.12	0.273710692
Ca-dependent events	14	2.14	0.262564103
Calmodulin induced events	14	2.14	0.262564103
CaM pathway	14	2.14	0.262564103
Cell-extracellular matrix interactions	15	2.2	0.254184397
PLCG1 events in ERBB2 signaling	18	2.23	0.252121212
DARPP-32 events	12	2.26	0.249756098
DAG and IP3 signaling	15	2.29	0.247017544
PLC-γ1 signaling	15	2.29	0.247017544
Amyloids	18	2.33	0.250980392
Telomere Maintenance	31	2.46	0.192688172
RNA polymerase I promoter opening	18	2.65	0.131282051
Chromosome maintenance	53	2.75	0.1024
Meiotic synapsis	24	2.88	0.077575758
Deposition of new CENPA-containing nucleosomes at the centromere	21	2.88	0.077575758
Nucleosome assembly	21	2.88	0.077575758
Packaging of telomere ends	12	3.09	0.044912281
Striated muscle contraction	21	4.76	0
Smooth muscle contraction	19	6.13	0
Muscle contraction	36	7.25	0

**Table 2:** Summary of the principal topological parameters estimated for the *de novo* reconstructed network.

Topological parameters	Heart network	Vessels network
Average clustering coefficient	0.195	0.234
Connected components	237	86
Avg. number of neighbors	6.329	15.611
Network radius	1	1
Network diameter	36	16
Network centralization	0.020	0.036
Network density	0.002	0.005
Network heterogeneity	1.198	1.183
