# Supplementary material for: Systems Biology Approach to the Dissection of the Complexity of Regulatory Networks in the S. scrofa Cardiocirculatory System
Source: Int J Mol Sci. 2013 Nov 21;14(11):23160–87. doi: 10.3390/ijms141123160 (PMC3856112; doi:10.3390/ijms141123160)
Supplement: Supplementary file 1 [file ijms-14-23160-s001.pdf]

Supplementary Information

Figure S1. COA analysis results. Bigger dot are samples, while smaller are genes. Red dot are heart, while blue, green and yellow are artery, veins and pulmonary artery. Black dots are leaflets. Light grey dots (**left**) are genes which expression correlate with heart samples, light grey dots (**right**) are genes which expression correlate with vessels and dark grey (**central**) are genes which expression does not be associated to specific samples and that are represented in the Figure 4 as pink dots.

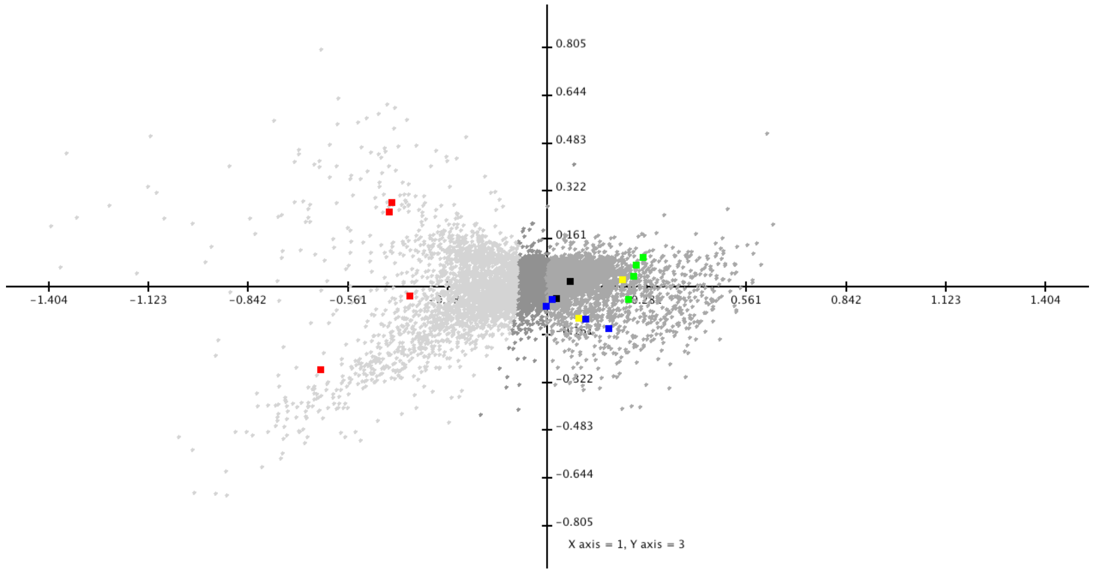

Figure S2. Probe design description to identify the exact 3'-ends of miRNAs. (**A**) Hairpin of the ENSSSCT00000020014/ssc-mir-136 where the blue rectangle represents the 3p arm and the red one the 5p arm. Black arrows show 0 position respectively in the 3p and 5p arms; green arrows show the ssc-mir-136 termini of the sequence deposited in the miRBase database; yellow arrows show the end positions we find for the miRNA that mature from the described hairpin. Braces below the hairpin indicate sequences complementary to the shifted probes of the −1, 1 and 14 regions respect to the 0 position; (**B**) Fluorescence intensity of each shifted probe. Blue bars show fluorescence of the probes in the 3p arm of the hairpin while red bars the one of 5p arm. All probes complementary to the sequence in the 3p hairpin arm have the same fluorescence intensity while those complementary to the 5p hairpin arm have a peak in the 13–16 region with the probe 14 corresponding to the 3'-end of the miRNA that presents the highest fluorescence intensity; (**C**) Sequences of the shifted probes complementary to the sequence of the 5p hairpin's arm. Sequences are characterized by a common spacer from the microarray surface, the specific sequence (orange) and a d(T) stretch useful for the RAKE experiments (detector). Only miRNA perfectly matched to the specific sequence will be extended by Klenow polymerase in the presence of only biotinilated-dATP. Background probes were chosen between all probes but 13–16.

A.

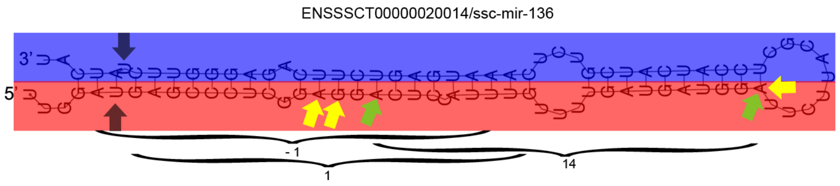

Figure S2. *Cont.*

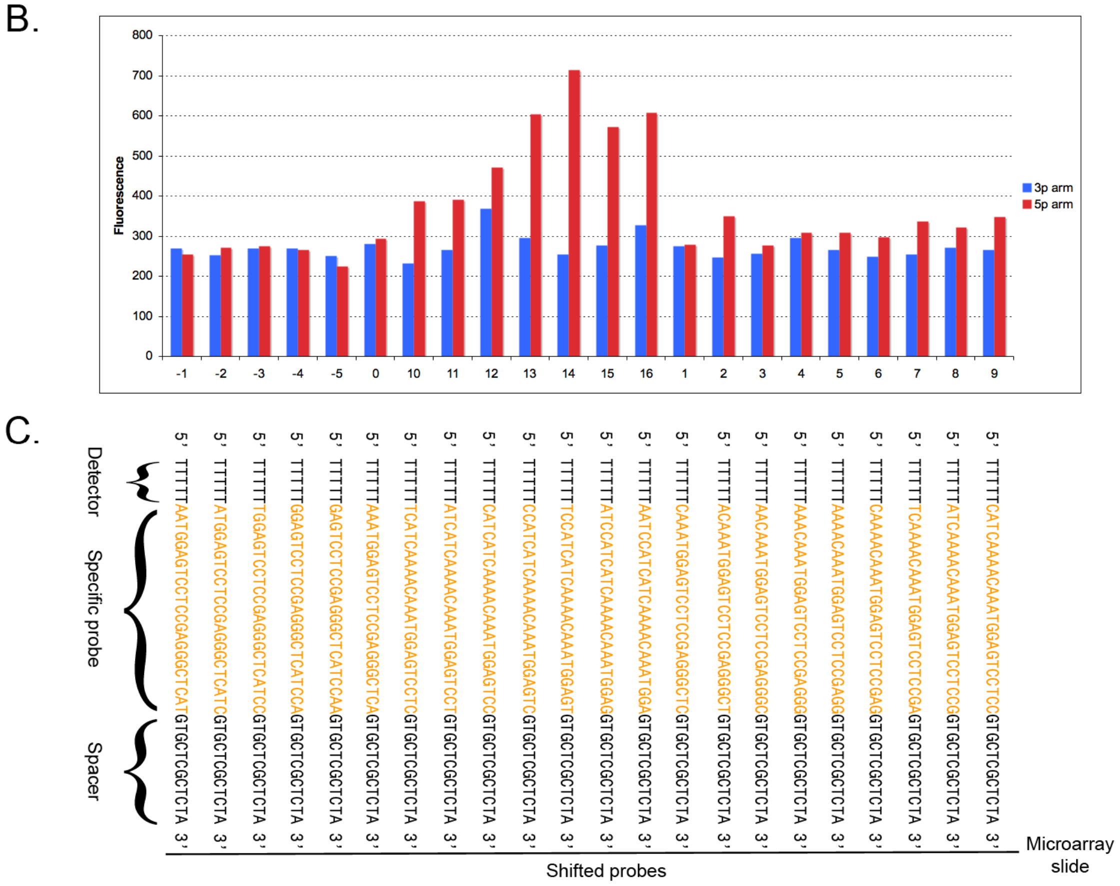

© 2013 by the authors; licensee MDPI, Basel, Switzerland. This article is an open-access article distributed under the terms and conditions of the Creative Commons Attribution license (http://creativecommons.org/licenses/by/3.0/).
